# Amphetamine Self-Administration and Its Extinction Alter the 5-HT_1B_ Receptor Protein Levels in Designated Structures of the Rat Brain

**DOI:** 10.1007/s12640-018-9950-y

**Published:** 2018-08-30

**Authors:** Joanna Miszkiel, Joanna Jastrzębska, Małgorzata Filip, Edmund Przegaliński

**Affiliations:** Department of Drug Addiction Pharmacology, Institute of Pharmacology, Polish Academy of Sciences, Smętna 12, 31-343 Kraków, Poland

**Keywords:** Serotonin (5-HT)_1B_ receptor expression, Immunohistochemistry, Amphetamine addiction, Amphetamine self-administration, Extinction training, Yoked procedure

## Abstract

Manipulation of the serotonin (5-HT)_1B_ receptors can modify the behavioral effects of amphetamine including its reinforcing properties. Focus of this study was to examine changes in 5-HT_1B_ receptor protein expression in several brain structures linked to substance drug disorder in different stages of amphetamine addiction—single session of amphetamine self-administration, 20 consecutive days of amphetamine self-administration, and 3 and 14 days of extinction from chronic drug intake. “Yoked” procedure was employed to set apart pharmacological and motivational effects of amphetamine intoxication. Immunohistofluorescence was performed on brain slices containing the following regions: nucleus accumbens (NAc) shell and core, globus pallidum (GP) lateral and ventral, hippocampus (HIP), substantia nigra (SN), and ventral tegmental area (VTA). Single amphetamine session decreased the amount of 5-HT_1B_ receptors in SN, VTA, and HIP in active and yoked rats. On the contrary, 20 days of chronic amphetamine exposure triggered elevation of 5-HT_1B_ receptors exclusively in animals that voluntarily administered the drug in NAc core, GP ventral, and HIP. Furthermore, 14-day (but not 3-day) extinction from amphetamine increased the 5-HT_1B_ receptor expression in ventral and lateral GP, HIP, and SN. This study is the first to demonstrate that exposure to amphetamine and its extinction alter the expression of 5-HT_1B_ receptors in various rat brain regions, and those changes seem to be transient and region specific. Importantly, since increased expression of 5-HT_1B_ receptor after chronic amphetamine self-administration was limited only to active group of animals, we suggest that 5-HT_1B_ receptor is linked to motivational aspect of addiction.

## Introduction

Serotonin (5-HT) transmission originates in raphe nuclei and spreads throughout the brain innervating almost all its parts (Parent et al. 1981; Steinbusch 1981). Abundance of the 5-HTergic connections, signaling via 14 different 5-HT receptors, makes this monoamine perfectly position to mediate many behavioral function, as well as to be responsible for maintaining a homeostasis of the system. Malfunction of this circuit can lead to many pathological states, among others, depression, schizophrenia, autism, or substance use disorders (Hoyer et al. 2002; Filip and Bader 2009). In fact, several data showed that among 5-HT receptors, 5-HT_1B_ ones seem to be especially promising in addiction research. Being negatively coupled to adenylyl cyclase and classified as both pre- and postsynaptic auto- and heteroreceptors, 5-HT_1B_ receptors exert inhibitory role on the release of several neurotransmitters including glutamate, GABA, and dopamine (Adell et al. 2001; Hoyer et al. 2002; Sari 2004). Also, their presence (protein, mRNA, or both) was confirmed in the brain areas that are crucial to reward system (nucleus accumbens (NAc), ventral tegmental area (VTA), hippocampus (HIP), globus pallidus (GP)) as well as are associated with drug-induced hyperlocomotion (substantia nigra (SN)) (Pazos and Palacios 1985; Bruinvels et al. 1993; Sari et al. 1997, 1999). A part of the preferential location, several pharmacological data showed that they indeed play significant role in psychostimulant drug addiction such as amphetamine; namely, mice lacking the 5-HT_1B_ receptor exhibited increased sensitivity to amphetamine measured as increased in locomotor activity (Bronsert et al. 2001). Interestingly, when these receptors were pharmacologically stimulated, not only they enhanced amphetamine-induced hyperlocomotion (Papla et al. 2002) but also facilitated the development of sensitization (Przegalinski et al. 2001) and decreased the number of lever presses in fixed and progressive ratio schedule (Fletcher et al. 2002; Miszkiel et al. 2012) in amphetamine self-administration model. Moreover, in the latter animal paradigm, administration of 5-HT_1B_ receptors antagonist attenuated the amphetamine-evoke seeking behavior (Miszkiel et al. 2012). However, there is still little known about molecular background of the 5-HT_1B_ receptor involvement in stages of amphetamine addiction.

On the other hand, there is a small body of literature indicating that mentioned receptors undergo time-dependent bidirectional alternation during the abstinence from chronic self-administration of another psychostimulant—cocaine (O’Dell et al. 2006). Furthermore, quantitative autoradiography indicated that after 5-day withdrawal from subchronic cocaine administration, 5-HT_1B_ receptors were upregulated in numerous brain structures of rat (Przegaliński et al. 2003). Although amphetamine and cocaine belong to the same group of psychostimulant drugs, they display several discrepancies in regard to 5-HT_1B_ receptor mediated effects. For instance, pharmacological stimulation of 5-HT_1B_ receptors increases reinforcing effects of cocaine (Parsons et al. 1998; Filip et al. 2002; Przegaliński et al. 2007; Pentkowski et al. 2009, 2012) but not of amphetamine (Fletcher and Korth 1999; Fletcher et al. 2002; Miszkiel et al. 2012). Moreover, even though 5-HT_1B_ receptor blockade attenuates cocaine (Przegaliński et al. 2008) and amphetamine-seeking behavior (Miszkiel and Przegaliński 2013), activation of those receptors decreases or has no effect on reinstatement of cocaine (Pentkowski et al. 2014) and amphetamine (Miszkiel and Przegaliński 2013), respectively. Keeping above in mind, it seems crucial to further investigate and eventually clarify the role of 5-HT_1B_ receptors in the drug addiction cycle. Therefore, this study was designed to determine the potential alternations in 5-HT_1B_ receptor protein level across different aspects of amphetamine addiction in rats.

## Materials and Methods

### Animals

Male Wistar rats (250–270 g) delivered by the licensed breeders (Charles River, Germany) were used in this experiment. Animals were housed individually in standard plastic rodent cages in a room maintained at 21 ± 1 °C and 40–50% humidity under a 12-h light-dark cycle (lights on at 6.00 a.m.). Rats were water restricted during initial lever-pressing training sessions and first 5 days of amphetamine self-administration. At other times, water was ad libitum. Access to food was unlimited throughout all experiment. All procedures were conducted during the light phase of the light-dark cycle. The experimental procedures were carried out in accordance with the European Directive 2010/63/EU and were approved by the Bioethical Committee at the Institute of Pharmacology, Polish Academy of Sciences, Krakow. Animals were drug-naive.

### Behavioral Experiments

#### Drugs

d-amphetamine sulfate (Sigma-Aldrich, USA) was dissolved in sterile 0.9% NaCl and given intravenously (0.1 ml/infusion).

#### Initial Lever-Press Training and Catheter Implantation

After 7-day initial habituation period, animals were water deprived for 18 h and then trained to lever press in 2-h daily sessions under the fixed ratio schedule (FR1) of water reinforcement for 2 to 5 days. Two days following the lever-press training and free access to water, the rats were implanted with a silastic catheter in the external jugular vein, as described previously (Filip et al. 2005). After surgery, the catheters were flushed daily with 0.1 ml of a heparinized saline solution (70 U/ml, Biochemie, Austria) and 0.1 ml of a cephazol in solution (10 mg/ml, Biochemie GmbH, Austria). No problems with catheter patency were reported in the tested rats. The rats were allowed 5 to 8 days to recover from surgery before the start of the experiments.

#### Maintenance of Amphetamine Self-Administration

After recovery, animals were again water restricted and placed in operant chambers to press lever for water for one 2-h session. Self-administration experiment begun on the following day, and rats were divided into separate groups (*n* = 6 each) and trained to press the lever for amphetamine reinforcement during 2-h daily sessions performed 6 days/week (for details see Miszkiel et al. 2012). Briefly, once placed in operant chamber, animals had to choose to press one of two presented levers. Each press (FR1) on the “active” lever resulted in a 5-s infusion of amphetamine (0.12 mg/kg/infusion), whereas pressing the “inactive” lever had no consequences. After 5 days during which animals reached a stable responding on the active lever, the number of presses that was required to achieve amphetamine injection was increased to FR3, and after subsequent 5 days, was increased again to FR5. Rats were allowed to remain on this schedule for 5 days to reach stable responding. Subsequently, amphetamine dose was reduced to 0.06 mg/kg/infusion, and self-administration was continued under FR5 for another 5 days. During the final 5 days, the number of active lever presses and the number of received infusions of amphetamine were stable and were not different between sessions by more than 10%; the number of inactive levers was no more than 50%. The number of daily amphetamine infusions was at least 10.

**Scheme 1:**
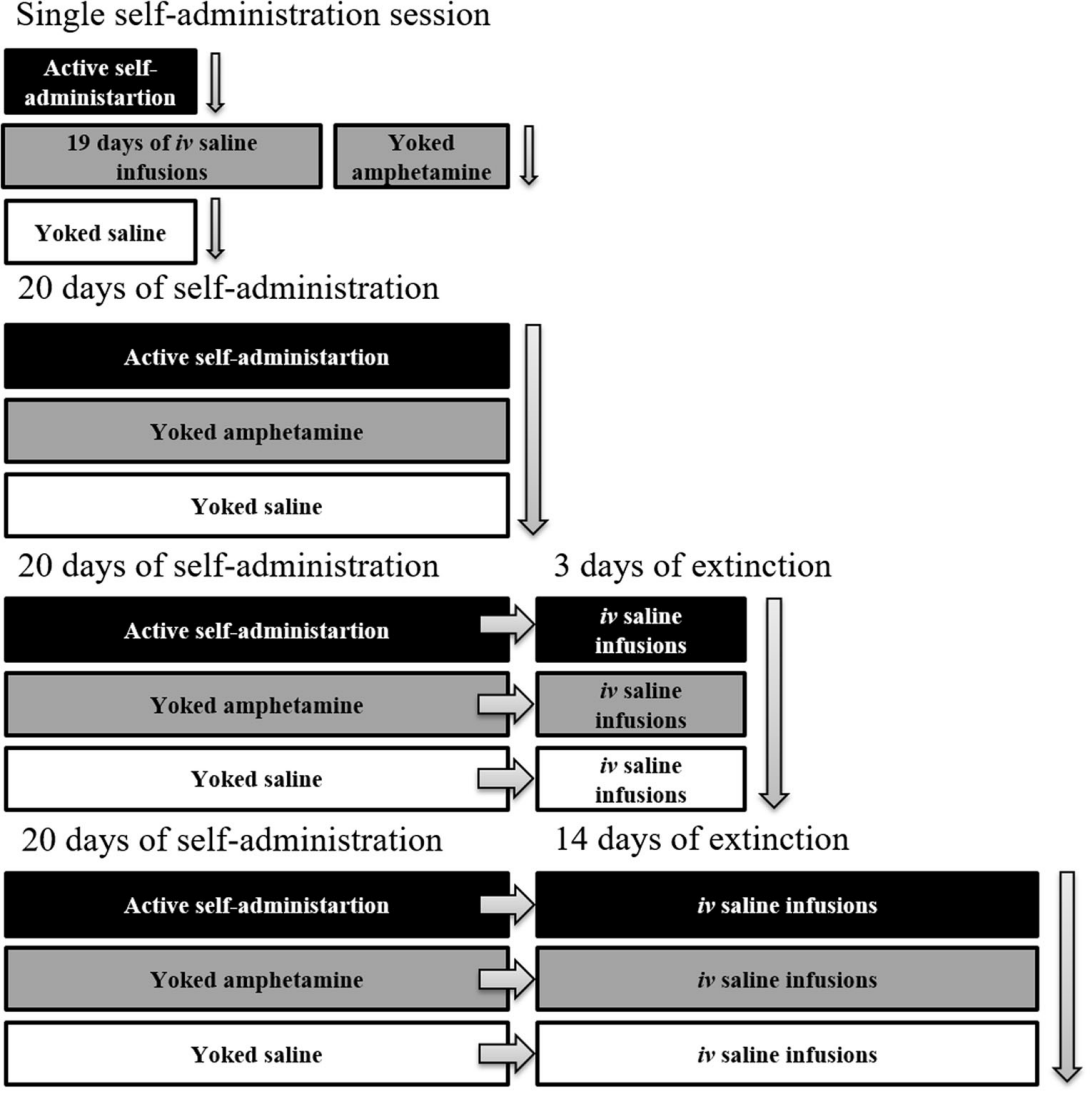
Experimental design for behavioral experiments. Vertical arrows represent time when appropriate group of animals were sacrificed

The animals actively administering amphetamine and corresponding “yoked” amphetamine or saline groups (for “yoked” procedure see below) were sacrificed immediately following the final 20th amphetamine self-administration session (see Scheme 1).

#### Single Amphetamine Self-Administration Session

After convalescence period, another group of animals were water deprived and reintroduced to operant chambers to press lever for water for one session. Next day, rats were allowed to self-administer amphetamine in self-administration operant chambers for one 2-h session under FR1 schedule. Every active lever pressing resulted in a 5-s delivery of amphetamine (0.06 mg/kg/infusion), and inactive lever pressing had no effect.

Animals actively administering amphetamine and corresponding “yoked” saline group were sacrificed immediately after the session (for “yoked” procedure see below). “Yoked” amphetamine rats were first introduced to 19 sessions during which they received passive saline injections in the same manner as active animal self-administered amphetamine. On the 20th final session, “yoked” amphetamine animals were passively administered amphetamine injections and were sacrificed instantly after.

#### Extinction Training

After 20 days of amphetamine self-administration, the rats underwent the extinction paradigm. During 2-h extinction session, animals were placed in the operant chambers described above and allowed to lever press (FR5); however, amphetamine was no longer present, and active lever presses resulted in saline delivery instead. Like in the maintenance, pressing the “inactive” lever had no consequences.

The animals actively administering amphetamine and corresponding “yoked” amphetamine or saline groups (for “yoked” procedure see below) were sacrificed upon completion of third or 14th extinction session (see Scheme 1).

#### “Yoked” Procedure

To distinguish between the pharmacological and motivational effects of amphetamine intake, “yoked” procedure was used for all experiments employed here. In this procedure, each rat actively self-administering amphetamine (or saline during extinction phase) has been assigned two rats that were passively receiving either amphetamine (or saline during extinction phase) or its vehicle in the same amount and manner as the active animal. Lever pressing by the “yoked” rats was recorded but had no programmed consequences.

#### Tissue Preparation

Immediately after the appropriate experimental sessions, animals (*n* = 6 group) were injected with pentobarbital (133.3 mg/kg, *i.p.,* Morbital, Biowet, Puławy, Poland) and perfused intracardially with a solution of 4% paraformaldehyde in 100-Mm phosphate buffer (pH = 7.4). The brains were immersed in the same fixative for 12 h. Then, tissues were permeated in 10% *w*/*v* sucrose at 4–8 °C for 7 days followed by 30% w/v sucrose for no less than 48 h.

### Immunohistochemistry Analyses

#### Brain Section Preparation

**Scheme 2:**
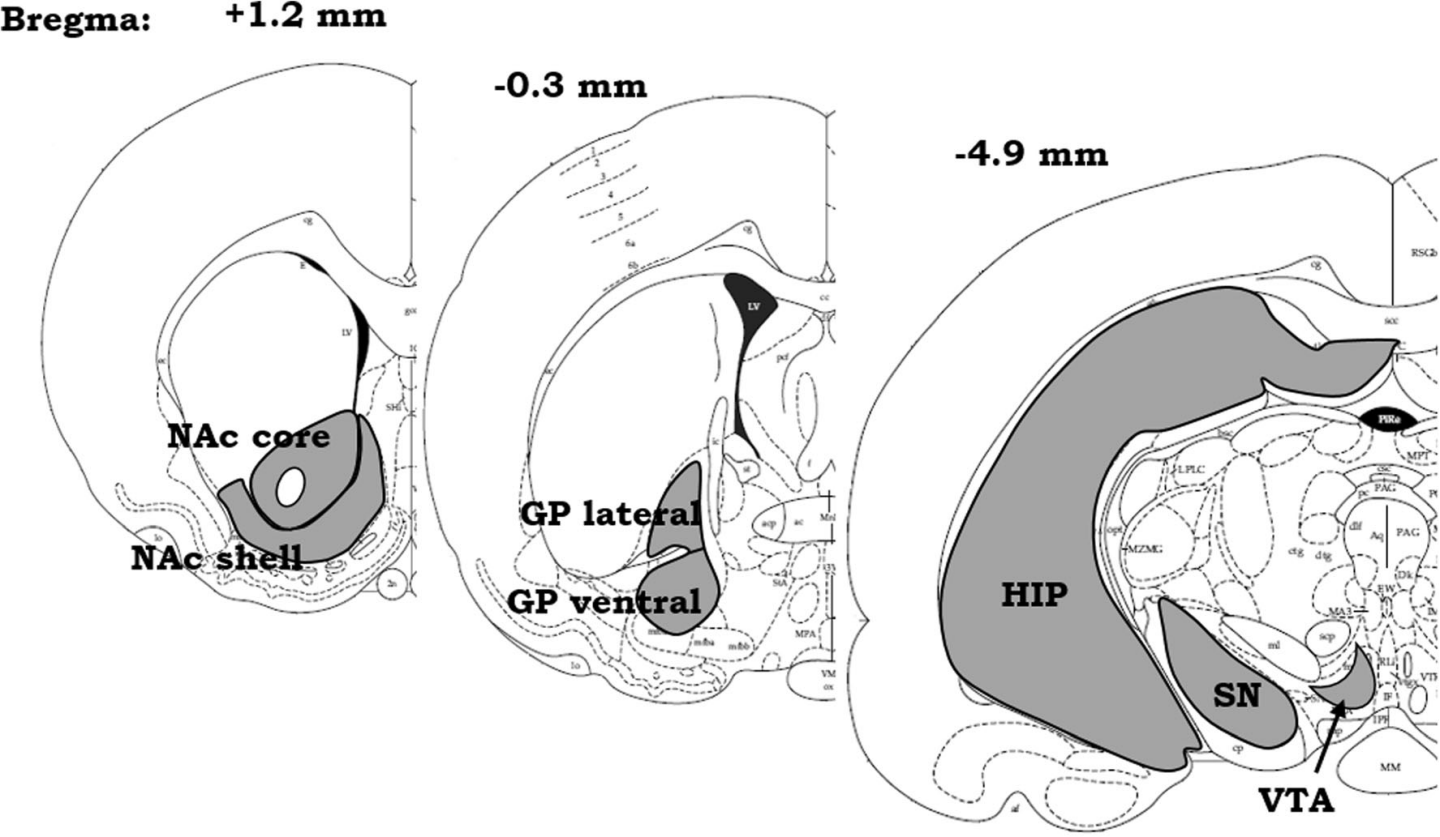
Scheme represents coronal brain structures: nucleus accumbens shell (NAc shell) and core (NAc core), globus pallidum lateral (GP lateral) and ventral (GP ventral), hippocampus (HIP), substantia nigra (SN), and ventral tegmental area (VTA) and their stereotactic coordinates according to the The Rat Brain Atlas (Paxinos and Watson 1998)

The brains were deeply frozen on dry ice, cut into 14-μm coronal sections on a cryostat (Leica Microsystems, Nussloch, Germany), and kept at − 22 °C until processed for immunohistochemistry. Stereotactic coordinates for the following brain structures: NAc shell and core, GP lateral and ventral, HIP, SN, and VTA, were determined and selected based on The Rat Brain Atlas (Paxinos and Watson 1998) (see Scheme 2). Brain slices with corresponding brain region(s) were mounted on gelled glass slides, in such a way that each slide housed all selected brain areas of one animal.

#### Protocol

Rat brain sections were rinsed with 100-mM PBS buffer (pH = 7.4) followed by the 30-min permeabilization in PBS buffer containing 0.1% Triton X-100 at room temperature. Afterwards, brain slices were incubated in Odyssey Blocking Buffer (OBB, Li-COR Biosciences, Cambridge, UK) for 1 h at room temperature. 5-HT_1B_ primary anybody (rabbit, polyclonal; Santa Cruz; sc-1460-R) was diluted to final concentration of 1:300 in OBB containing 0.1% Tween 20 and incubated on a specimen overnight at 4 °C. On the next day, brain slices were washed in PBS containing 0.1% Tween 20 (4 × 5 min), and secondary antibody goat anti-rabbit (IRDye® 680CW; Li-COR Biosciences, Cambridge, UK) was applied and incubated at room temperature for 1 h. Slices were then washed in PBS containing 0.1% Tween 20 (4 × 5 min) and PBS (1 × 5 min) and left to dry. Fluorescence was detected using the Odyssey® Infrared Imaging System (21-μm resolution, 1-mm offset with high quality) using 700-nm channel. The integrated intensities were determined with the associated Odyssey software. Each section was prescanned at different intensity settings on the Odyssey Classic Infrared Imaging System. Channel sensitivity was optimized for each set of stained sections, and channel intensity varied from 2 to 5. The latter allows detection of nonspecific background signals from the sample and permits gross localization of the cerebral tissue. Regions of interest were defined by comparison to The Rat Brain Atlas (Paxinos and Watson 1998) and marked using the in-software drawing tool. Data expressed as fluorescence relative units were later exported, analyzed, and normalized to saline-treated animals.

### Statistical Analyses

Behavioral data were analyzed by a two-way analysis of variance (ANOVA) for repeated measures, whereas for the immunohistochemical assays, one-way ANOVA was used. If the effect was significant, a post hoc Newman-Keuls’ test was applied to evaluate statistically significant differences between the treatment groups. All data are presented as the mean ± SEM, and in all cases, *p* value less than 0.05 was considered significant.

## Results

### Behavioral Studies

Animals that were introduced to single 2-h amphetamine session scored 38.4 ± 9.4 lever presses on the active lever and 6.7 ± 3.1 on the inactive, receiving on average 1.6 ± 0.1 mg/kg amphetamine per rat.

**Fig. 1:**
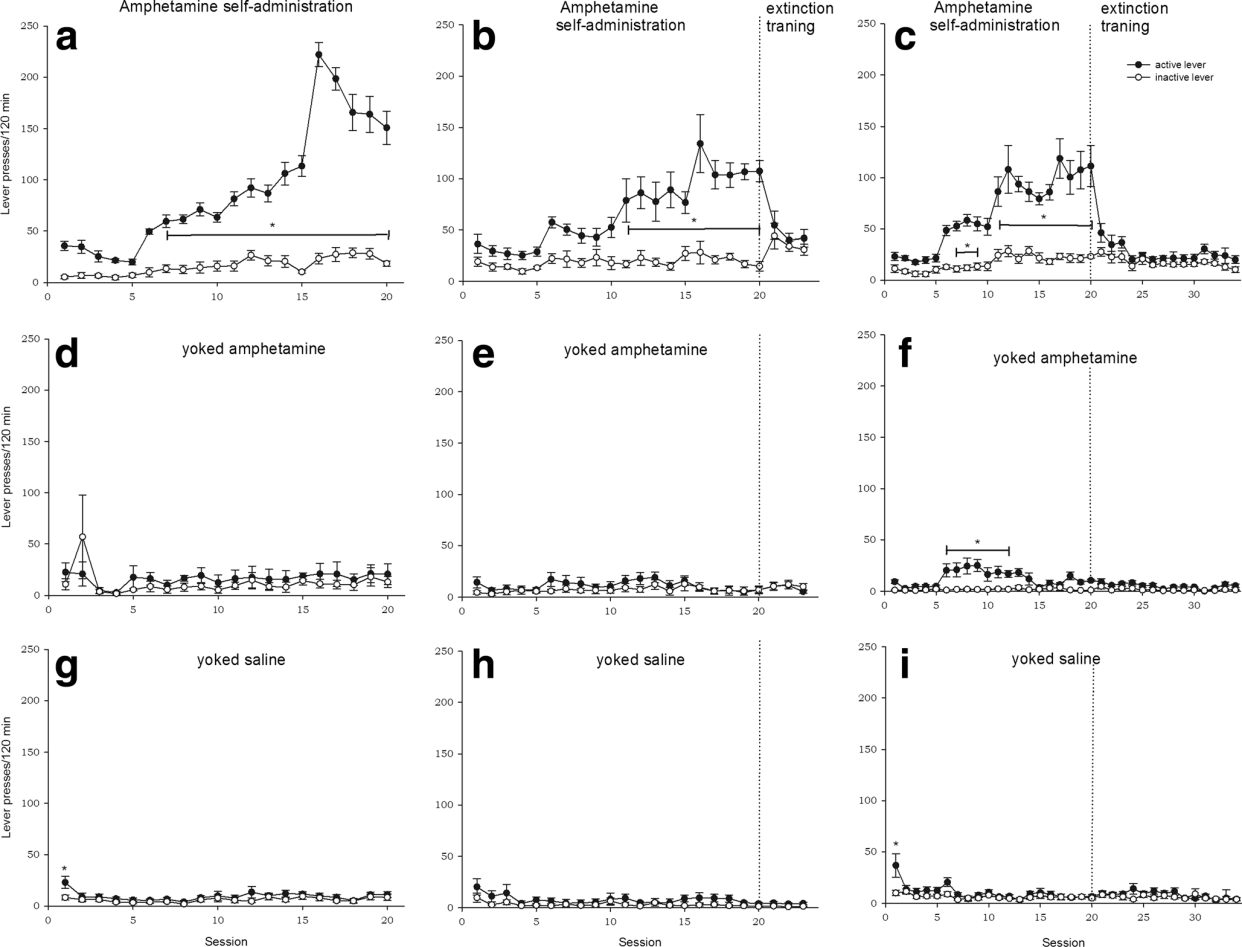
The number of active and inactive lever presses in rats that acquired and maintained amphetamine (0.12–0.6 mg/kg/infusion) self-administration (**a**), 3-day extinction training (**b**), and 14-day extinction training (**c**) with their yoked controls that passively received amphetamine (**d**–**f**) or saline (**g**–**i**). Data are presented as the mean ± SEM from 6 rats/group. * indicates the statistically significant difference between active and inactive lever presses of minimum value *p* < 0.05

Rats from remaining experiments that were allowed to self-administer amphetamine for 20 days showed stable level of response (FR5) during last five amphetamine sessions. Averaged number of active lever presses during the last 5 days of amphetamine (0.06 mg/kg) self-administration was 128 ± 11 on the active and 22 ± 1 on the inactive lever, while the number of infusions was 17 ± 2. Throughout the 20 days of amphetamine self-administration, rats earned on averaged 33 ± 1.4 mg/kg amphetamine per rat. The two-way ANOVA for repeated measures showed that from the day 11 to the end of the maintenance phase, the number of active lever presses was statistically greater than the number of the inactive lever presses (*p* < 0.05; Fig. 1a–c). During the extinction phase, amphetamine was no longer available, which resulted in a decrease in active lever presses; and from the 21^st^ to the 23^rd^ or 34^th^ experimental sessions, the difference between responses to the active versus inactive lever was no longer significant (Fig. 1b, c, respectively).

In the yoked amphetamine groups, animals received the same amount of amphetamine at the same time as the animals that learned to self-inject amphetamine, without developing the preference towards active lever. Number of active lever presses did not differ from inactive lever presses neither during the maintenance nor the extinction phase (Fig. 1d, e) except the yoked amphetamine animals from the group that underwent 14 days of extinction. The latter group exhibited small, but yet significant (*p* < 0.05), increase in active lever presses from day 6 to day 12 (Fig. 1f). Rats in yoked saline groups received saline infusions in the same manner as paired active rat self-administer amphetamine. Except for the first day, the difference between responses to the active versus inactive lever was not significant (Fig. 1g–i).

### Immunohistochemical Staining

The 5-HT_1B_ receptor protein expression was assessed in the following brain regions: NAc shell and core, GP dorsal and ventral, HIP, SN, and VTA. Protein expression was examined in four time points—after single 2-h amphetamine session, after 20 days of drug self-administration, and after 3 and 14 days of extinction from amphetamine.

### Single 2-h Amphetamine Session

**Fig. 2:**
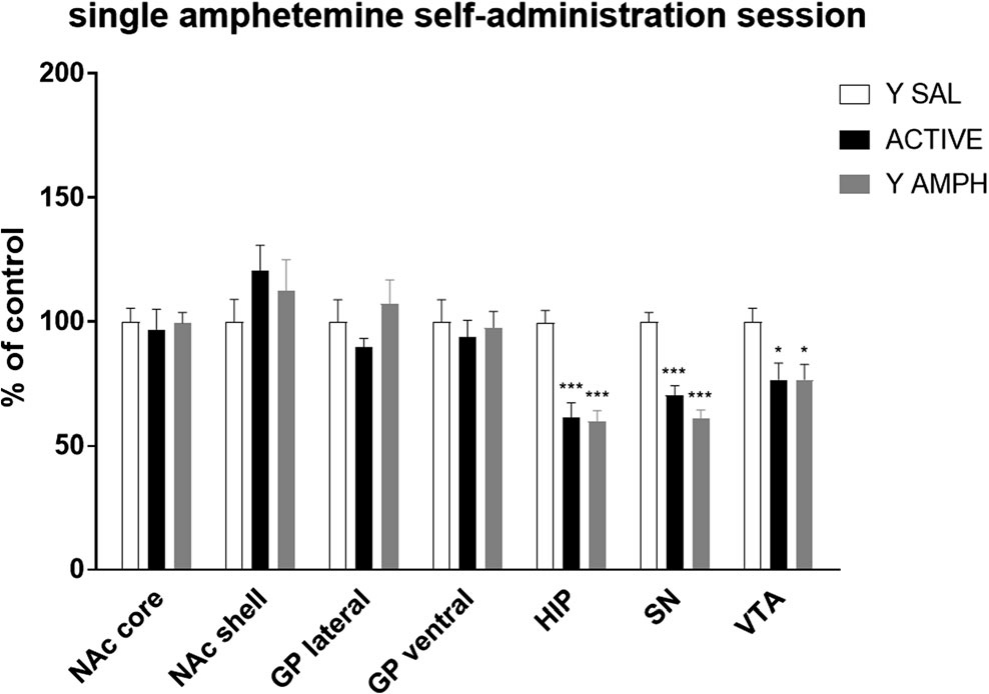
Effect of single amphetamine self-administration session on the expression of 5-HT_1B_ receptors in nucleus accumbens shell (NAc shell) and core (NAc core), globus pallidum lateral (GP lateral) and ventral (GP ventral), hippocampus (HIP), substantia nigra (SN), and ventral tegmental area (VTA) in animals voluntarily taking the drug (ACTIVE). Control groups of rats passively receiving amphetamine (Y AMPH) or saline (Y SAL) were generated by “yoked” procedure. Data were normalized to saline-treated animals (% of yoked saline) and are shown as the mean (± SEM) of 6 subjects/group. Data were analyzed using one-way ANOVA and the post hoc Newman-Keuls’ test. *p < 0.05 and ***p < 0.001 versus yoked saline within that brain structure

According to one-way ANOVA analysis, animals that underwent single 2-h amphetamine session showed a significant effect for 5-HT_1B_ receptor immunofluorescence in HIP (*F*_(2, 15)_ = 19.735; *p* < 0.001), SN (*F*_(2, 15)_ = 28.590; *p* < 0.001), and VTA (*F*_(2, 15)_ = 4.826; *p* < 0.05) (Fig. 2). Further post hoc analysis revealed that vast, up to 40%, decrease in 5-HT_1B_ receptor protein expressions in HIP and SN in both amphetamine-treated groups was significantly lower (*p* < 0.001) than protein levels of this receptor in yoked saline animals. Smaller (25%), but also significant, reduction in 5-HT_1B_ receptor expression was observed in active and yoked amphetamine animals (*p* < 0.05) in comparison to saline control group in VTA.

### Chronic Amphetamine Self-Administration

**Fig. 3:**
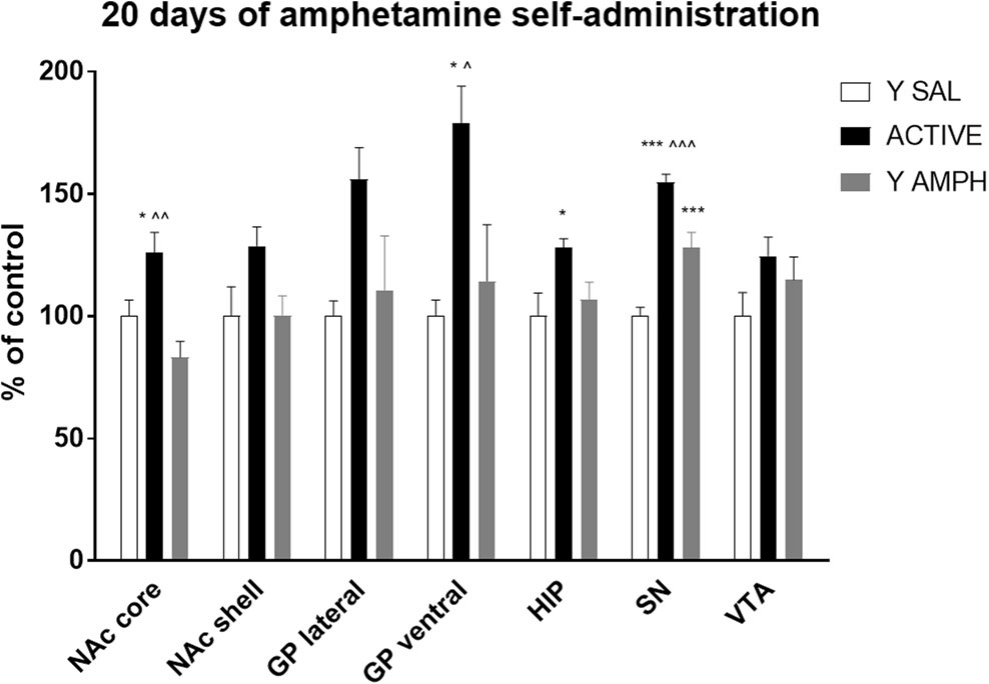
Effect of 20-day amphetamine self-administration on the expression of 5-HT_1B_ receptors. For more details see Fig. 2. *p < 0.05, ***p < 0.001 versus yoked saline within that brain structure. ^*p* < 0.05, ^^*p* < 0.01, and ^^^*p* < 0.001 versus yoked amphetamine within that brain structure

The effect of amphetamine self-administration on 5-HT_1B_ receptor expression in rat brain structures is shown in Fig. 3. One-way ANOVA shown significant changes in NAc core (*F*_(2, 15)_ = 9.109; *p* < 0.01), lateral (*F*_(2, 15)_ = 3.738; *p* < 0.05), and ventral (*F*_(2, 15)_ = 6.533; *p* < 0.01) parts of GP, HIP (*F*_(2, 15)_ = 4.119; *p* < 0.05), and SN (*F*_(2, 15)_ = 38.158; *p* < 0.001). A similar but insignificant trend was observed for NAc shell (*F*_(2, 15)_ = 3.030; *p* = 0.078). Newman-Keuls’ post hoc analysis determined that in NAc core, level of the 5-HT_1B_ receptor protein in animals actively administering amphetamine was elevated (25%) and significantly different from levels found in yoked saline (*p* < 0.05) and amphetamine (*p* < 0.01) rats. A robust increase (55 and 80%) of examine receptors in animals with self-administration history versus yoked saline rats was also noted in in lateral and ventral parts of GP. However, according to the post hoc test employed, only the latter increase was significant (*p* = 0.05, *p* < 0.01, respectively). Moreover, this alternation was also significant in relation to the group of rats passively given the drug (*p* < 0.05). In the HIP, a significant (*p* < 0.05) 30% enhancement in immunofluorescence signal was seen in rats actively taking amphetamine in comparison to the yoked saline group. In SN increase in 5-HT_1B_ receptor expressions was observed in both active (55%; *p* < 0.001) and yoked amphetamine (30%; *p* < 0.001) rats versus control. Moreover, post hoc test showed a significant difference between animals actively taking and passively receiving amphetamine injections (*p* < 0.001).

### Short (3-Day) Extinction from Amphetamine Self-Administration

**Fig. 4:**
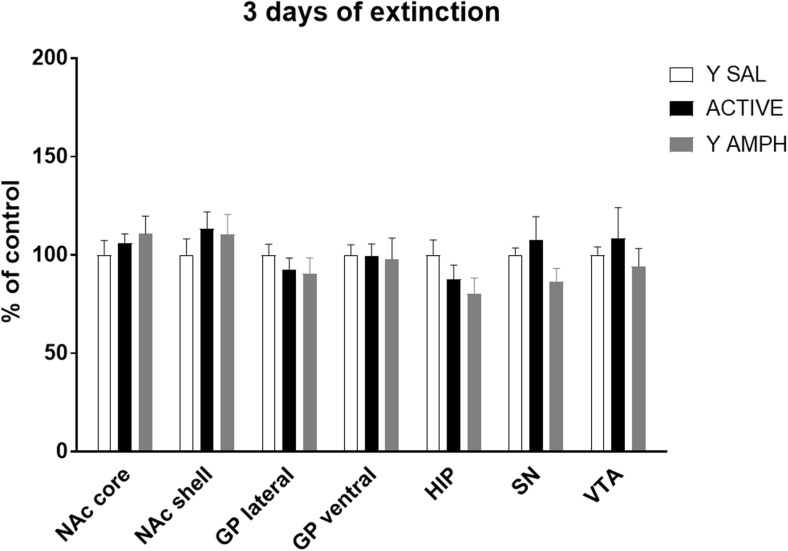
Effect of 3-day extinction from chronic amphetamine self-administration on the expression of 5-HT_1B_ receptors. For more details see Fig. 2

No changes in receptor expression were observed at this time point (Fig. 4).

### Long (14-Day) Extinction from Amphetamine Self-Administration

**Fig. 5:**
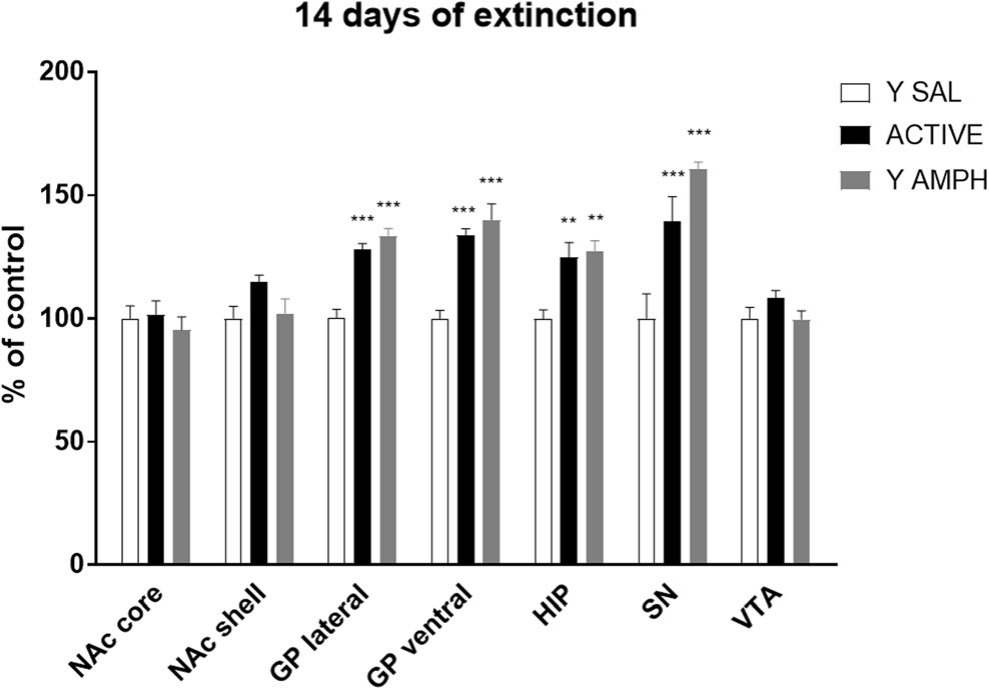
Effect of 14-day extinction from chronic amphetamine self-administration on the expression of 5-HT_1B_ receptors. For more details see Fig. 2. **p < 0.01 and ***p < 0.001 versus yoked saline within that brain structure

As shown on Fig. 5, following 14-day extinction training, a one-way ANOVA showed a significant effect in 5-HT_1B_ receptor protein expressions in GP lateral (*F*_(2, 15)_ = 36.095, *p* < 0.001) and ventral (34%) (*F*_(2, 15)_ = 23.819; *p* < 0.001), HIP (25%) (*F*_(2, 15)_ = 9.769; *p* < 0.01), and SN (*F*_(2, 15)_ = 13.482; *p* < 0.001) in rats with a history of amphetamine self-administration and their yoked amphetamine controls. After the post hoc test, significant (*p* < 0.001), 40% increase of the immunofluorescent signal was noted in animals with active and passive amphetamine intake history in lateral and ventral GP. Lower (25%) but significant (*p* < 0.01) rise in expression of 5-HT_1B_ receptors was observed in HIP also in both active and yoked amphetamine rats versus control group. The same trend was shown in SN, where 40 and 60% elevation in 5-HT_1B_ receptor protein was reported in rats previously voluntarily administered (*p* < 0.01) and passively received (*p* < 0.001) amphetamine, respectively.

## Discussion

Classical theory of receptor regulation explains that G protein-coupled receptors (GPCRs) can undergo internalization and/or downregulation when overstimulated or becomes upregulated in response to long-term receptor blockade or deprivation. This theory has been supported for numerous receptors including those for 5-HT ones. In general, various 5-HT receptors, for instance 5-HT_1B_, will downregulate when exposed to extensive 5-HT system activation (Fabre et al. 2000; van Oekelen et al. 2003) and upregulate when 5-HT neurotransmission system becomes impaired (Manrique et al. 1994; Compan et al. 1998).

A strong psychostimulant—amphetamine—primarily known to elevate the extracellular levels of DA and NE, also elevates the 5-HT concentration (for review see Faraone 2018). Interestingly, however, it was shown that this drug can alter 5-HT efflux in both directions, depending on administration pattern, time, and brain structure. Indeed, single amphetamine administration increased extracellular efflux of 5-HT in various brain structures (Parada et al. 1988; Kuczenski and Segal 1997; Salomon et al. 2006; Pum et al. 2007), but when this drug was administered repeatedly, global 5-HT concentration declined (McMillen et al. 1991), while local (for instance PFCx) levels of this monoamine remained intact (Salomon et al. 2006). Furthermore, when extracellular 5-HT was measured after withdrawal from chronic amphetamine treatment, Barr et al. (2010) reported initial decrease (measured 20 h after last injection) in 5-HT concentration in dentate gyrus that was no longer detected after 4 weeks of drug-free period. Interestingly, 4 days after repeated amphetamine administration, increased cortical extracellular 5-HT level was reported following p-chloroamphetamine administration (Salomon et al. 2006).

In light of the theory of receptor regulation, these 5-HT fluctuations in response to amphetamine administration may lead to adaptations on the receptor protein level. Indeed, our study demonstrated that this psychostimulant triggered changes in the 5-HT_1B_ receptor density that are similar to the amphetamine-induced changes in extracellular 5-HT described above and that alternations were dynamic, time, and structure dependent. To the best of our knowledge, there are no other studies regarding changes in 5-HT_1B_ receptor density following amphetamine administration that employed self-administration model. However, Bonhomme et al. (1995) using sensitization paradigm and quantitative autoradiographic analysis examined the expression of 5-HT_1B_ receptor changes in several brain structures on the days 1 and 15 after 6-day experimenter-administered amphetamine regime. Similar to our results obtained from yoked rats, they showed no changes in 5-H_1B_ receptor expression levels in medial PFCx, NAc, STR, SN, VTA, and medial raphe nuclei shortly after chronic amphetamine exposure. Interestingly, no changes were detected 2 weeks later, whereas we found an increase in 5-HT_1B_ protein levels at this time point. One possible explanation of the lack of receptor upregulation in sensitized animals is the difference in the housing condition that, in case of Bonhomme et al. study (1995), could have helped elevate the amphetamine withdrawal related stress/depression. Specifically, rats from our experiments were isolated throughout entire self-administration/extinction procedure, whereas animals from Bonhomme et al. (1995) studies were placed into individual cages only for the duration of the behavioral scoring. Lack of drug in the system due to the forced withdrawal period in both of the experiments most likely triggered the anxiety and depression-like states in all animals (Barr et al. 2010; Vuong et al. 2010; Li et al. 2014; Reinbold et al. 2014; Tu et al. 2014); however, the amphetamine-related withdrawal symptoms could have been less severe in animals which had contact with their littermates for the majority of time. In fact, recent study has shown that those drug-evoked withdrawal symptoms and depressive-like behaviors can be diminished when animals are provided with environmental enrichment (Hajheidari et al. 2015). Even though the housing condition employed by Bonhomme et al. (1995) do not resemble the environmental enrichment habitats per se, it is likely that it was enough to counterbalance the 5-HT_1B_ receptor upregulation observed in our experiment.

Another explanation to the elevated expression of the 5-HT_1B_ receptors after 2 weeks of amphetamine-free period could be that it was a result of their contribution to the incubation of craving (Pickens et al. 2011). Indeed, research has shown that seeking behavior increases in time-depended manner during the forced abstinence from drug of abuse, including psychostimulants such as cocaine (Grimm et al. 2001; Mead et al. 2007; Neisewander et al. 2000) and methamphetamine (Li et al. 2015; Venniro et al. 2017). Moreover, at least regarding to cocaine, this phenomenon was reported to be partially driven by 5-HT2C receptors (Swinford-Jackson et al. 2016). There is no evidence yet linking 5-HT_1B_ receptors with incubation of amphetamine craving; however, study showed that stimulation of this receptor attenuates cocaine-seeking behavior in a manner depended on length of abstinence (Pentkowski et al. 2014). With this in mind, the possibility that observed increase in 5-HT_1B_ expression protein in our study was related to incubation of amphetamine craving cannot be ruled out. Whether that lack of such increase reported by Bonhomme et al. (1995) was a consequence of the housing conditions or the passive drug administration needs to be determined.

As mentioned above, there is very little known about the effect of amphetamine on 5-HT_1B_ receptors; however, a few studies examined the effect of its derivate—3,4-methylenedioxymethamphetamine (MDMA) on these receptors. These studies showed that after the last dose of intermittent MDMA treatment, 5-HT_1B_ receptor mRNA was increased in several brain structures including STR (Kindlundh-Högberg et al. 2006), but that increase was undetected 24 h following the chronic drug administration (Sexton et al. 1999). Interestingly, at the latter time point, an increase in 5-HT_1B_ receptor binding sites in the STR was reported, regardless unchanged mRNA levels (Sexton et al. 1999). Transient and unequivocal alternations in levels of 5-HT_1B_ mRNA receptors were also observed after administration of another psychostimulant—cocaine. Acute administration of this drug had either no effect (Hoplight et al. 2007) or increase in the 5-HT_1B_ mRNA levels in STR (Neumaier et al. 2009). Passive chronic cocaine administration elevated 5-HT_1B_ mRNA (Hoplight et al. 2007), but when animals were self-administering the same drug, it had no effect on 5-HT_1B_ receptor levels (Neumaier et al. 2009). Furthermore, 5-day withdrawal from chronic passive cocaine exposure led to increased 5-HT_1B_ receptor levels in several brain structures including NAc shell and STR (Przegaliński et al. 2003), whereas 2-week forced abstinence from self-administration caused their downregulation in NAc shell and STR (Neumaier et al. 2009).

Similar changes in expression patterns have been observed for 5-HT_2A_ receptors after amphetamine administration; namely, McMillen et al. (1991) observed downregulation of 5-HT_2_ receptor mRNA in FCx after 7-day experimenter-administered amphetamine treatment. More recent studies examined the effect of escalating dose regimen of passively delivered amphetamine on 5-HT_2A_ receptor mRNA and reported the same direction of changes after 24 h from last amphetamine dose in the cortical areas including PFCx (Horner et al. 2011; Murray et al. 2014). Interestingly, at least in the latter structure, these changes seemed to be transient, since after 4 days of withdrawal 5-HT_2A_ mRNA was upregulated (Murray et al. 2014). Direction of changes may also be structure limited since 5-HT_2A_ mRNA was increased in NAc, STR, and HIP a day after the last drug injection (Horner et al. 2011). Taken together with the current findings, it appears likely that amphetamine transiently induces site specific changes to the expression of multiple 5-HT receptor subtypes. It is possible that those observed changes in 5-HT receptor expression is a system’s way of restoring the homeostasis; however, more studies need to be perform to better understand the phenomenon.

Another aspect of this paper was to examine differences between active and passive amphetamine administration. The most important finding from this study was that 20 days of chronic amphetamine exposure triggered elevation of 5-HT_1B_ receptors exclusively in animals that voluntarily administer the drug in the brain regions that are part of reward circuit and are involved in addiction-related behavior. In fact, the mesolimbic dopamine system that projects from VTA to the NAc was numerously reported to be essential for rewarding effects of drugs of abuse (Fibiger and Phillips 1986; Wise and Bozarth 1987; Koob 1992; Wise 1996; McBride et al. 1999; Pierce and Kumaresan 2006). Furthermore, the DA release into the core compartment of NAc is a necessary factor to initiate reward-related instrumental responses (Cardinal et al. 2002) as well as reward-associated learning (Scofield et al. 2016). On the other hand, one of the main outputs of NAc is ventral GP (Heimer and Wilson 1975), a brain region acting as hub for direct and indirect basal ganglia pathway converting limbic motivation signal into motor outputs (Mogenson et al. 1980; Mogenson and Yang 1991) and therefore controlling variety of behaviors, among others, goal-directed actions, decision making, and motivation (Gerfen et al. 1982; Albin et al. 1989; Deniau and Chevalier 1992; Smith et al. 2009; Gerfen and Surmeier 2011; Arimura et al. 2013; Calabresi et al. 2014). Finally, a structure that is also closely connected to the reward circuit is the HIP. It receives a vast dopaminergic input from VTA which has strong influence on learning and memory (Frey et al. 1990, 1991; Matthies et al. 1997; Lisman and Grace 2005; Granado et al. 2008) and also connects back with this structure through indirect loop via NAc and ventral GP (Legault and Wise 1999; Floresco et al. 2003; Lodge and Grace 2006). This way, HIP is well positioned to mediate between reward and limbic areas and to allow the initiation of the further addiction states: compulsive behavior and habit formation (Gerdeman et al. 2003; Everitt et al. 2008; Everitt and Robbins 2013; Everitt 2014). To the best of our knowledge, there is little to none known about the relationship between the 5-HT_1B_ receptors located in the NAc core, GP ventral, and HIP and amphetamine self-administration and goal-directed behaviors. Therefore, we find the result obtained from this study, indicating that 5-HT_1B_ receptors’ function(s), at least in above mentioned structures, is directly linked to cognitive processes of addiction and most likely plays a role in incentive drug taking, exciting, and important to the addiction field.

One way to explain this phenomenon could be that only active group was able to learn to anticipate the drug injection and/or its effects whereas others had diminished or abolished expectancy of the substance intake. Even though the passive drug administration has been proven to evoke rewarding effects (i.e., conditioning place preference), the absence of expectancy can make a difference, perhaps increasing the stressful responses instead. As the matter of fact, Dworkin et al. (1995) shown that passive cocaine injections cause higher mortality rate than willful drug self-administration in rats. At the same time, other possible explanation is the differential involvement of DA neurotransmission in animals that undergo active versus passive drug administration; however, data are unequivocal. For instance, one study demonstrated that extracellular levels of DA in the NAc were higher in rats that actively administered cocaine than in those that received the drug passively (Hemby et al. 1997). On the contrary, during amphetamine self-administration, short-term extinction and reinstatement, yoked (but not active) animals exhibited higher DA levels in NAc (Ranaldi et al. 1999). Also experimenter-administered heroin, but not active heroine administration, increased DA release in the latter structure (Hemby et al. 1995). Whether the observed alternation in 5-HT_1B_ receptor expression between active and passive amphetamine administration are evoked by the stress responses, differential DA involvement or not yet named contributors need to be determined; however, their link to incentive drug taking remains rather certain.

Parallel changes in 5-HT_1B_ receptor expression were reported in the remaining groups of animals, which is not unexpected. In the group of animals that were exposed to only single amphetamine session, even though only active rats were able to titer the amount and time of the drug delivery, this was the first time exposure to the drug for both, and observed changes might have been due to pharmacological result of amphetamine intake. As for the rodents that underwent amphetamine self-administration followed by 14-day extinction, it is likely that results are cause by lack of drug in the system and perhaps due to the effect of withdrawal-related stress or incubation of amphetamine craving (see above).

Both passive and active administration of amphetamine exerts powerful effect on numerous behavioral functions such as locomotor activity, aggression, mood, and reward circuits (Seiden et al. 1993) in which 5-HT_1B_ receptor engagement was also indicated (Sari 2004). Furthermore, direct relationship between these receptors and amphetamine-induced rewarding behaviors was also demonstrated. Precisely, in amphetamine self-administration model in rats, pharmacological stimulation of 5-HT_1B_ receptor decreased the number of lever presses in fixed (Miszkiel et al. 2012) and progressive ratio schedule (Fletcher et al. 2002). Interestingly, Miszkiel et al. (2012) concluded that observed reduction in lever presses and drug intake was due to behavioral disruption and not to increase of reward activity. Here, we showed that prolonged amphetamine exposure elevated 5-HT_1B_ receptor expression in numerous structures, including NAc core, which is part of a reward circuit. Therefore, it is possible that agonist-induced behavior observed by Miszkiel and others (Miszkiel et al. 2012) was caused by ceiling effect. Additionally, a motivational aspect of drug intake may be important. Previous data reported that the same agonist of 5-HT_1B_ receptors enhanced the locomotor hyperactivity induced by amphetamine in mice (Przegalinski et al. 2001), and 5-HT_1B_ receptor overexpression facilitated cocaine-induced locomotor activity in rats (Neumaier et al. 2002). However, it should be noted that in these studies, drugs were passively injected; and in the current study, the increase in 5-HT receptor protein was observed only in animals that administered the drug actively. The reason why active and passive amphetamine administration would lead to opposite behavioral effects is still yet to be determined. On the other hand, 2 weeks of amphetamine extinction led to overexpression of 5-HT_1B_ receptors regardless of animals’ drug history. As reported earlier (Miszkiel and Przegaliński 2013), pharmacological blockade of these receptors attenuated the amphetamine-evoke reinstatement. Therefore, taken together, it is plausible that by normalizing the 5-HT_1B_ activity, homeostasis would be restored, and drug seeking behavior could be diminished. However, it needs to be mentioned that even though 5-HT_1B_ receptors were elevated during the amphetamine self-administration, the same antagonist remained ineffective in decreasing the amphetamine intake or lever presses during the maintenance phase (Miszkiel et al. 2012).

Concluding, this is the first study to demonstrate that single amphetamine session, chronic amphetamine self-administration, and 14-day extinction from this drug alter the expression of 5-HT_1B_ receptors in various brain regions. Furthermore, increased expression of 5-HT_1B_ receptors after chronic drug intake was limited to the active group of animals, potentially linking 5-HT_1B_ receptor with motivation aspect of addiction. However, several questions regarding the expression pattern of these receptors remain unanswered and need further examination.
